# Women and children first: Delivering solutions in conflict-affected settings

**DOI:** 10.1371/journal.pgph.0004084

**Published:** 2025-01-08

**Authors:** Maria El Bizri, Etienne V. Langlois, Amy Reid, Rajat Khosla

**Affiliations:** Partnership for Maternal, Newborn, Child Health (PMNCH), World Health Organization (WHO), Geneva, Switzerland; PLOS: Public Library of Science, UNITED STATES OF AMERICA

Violent conflicts today are reported in every region of the world and often lasting for protracted periods of time. More than 45 armed conflicts are active in the Middle East and North Africa alone, over 35 in Africa, 21 in Asia, 7 in Europe, and 6 in Latin America [1]. These conflicts leave indelible marks on societies, yet their effects are neither evenly distributed nor universally endured, with women, newborns, and children being among the most vulnerable. Amid rising global instability, the imperative to protect the most vulnerable has never been more urgent.

The human toll of conflict is substantial. In 2023 alone, the United Nations recorded 33,443 civilian deaths in armed conflicts—a staggering figure likely underestimated due to inaccessible conflict zones and collapsed reporting mechanisms [2]. Among these, women accounted for four out of every ten fatalities, while children made up three out of ten [2]. In Gaza, between November 2023 and April 2024, 70% of those killed in civilian homes were women and children, and the most affected age group were children aged 0–4 [3]. In Ukraine, since February 2022, conflict-related violence had resulted in 30,457 civilian casualties, including 1,885 children [4]. These figures reveal the devastating human cost of armed conflict and the particular vulnerabilities of women and children caught in the crossfire.

Sexual and gender-based violence is compounded by these crises. Over 70% of women in conflict zones experience sexual violence—double the global average [5]. Tactics such as rape, forced pregnancy, and sexual slavery are used as weapons of war, with lasting repercussions for survivors and their communities. In Sudan, recent reports show that between April and December 2023 at least 118 cases of sexual violence were documented, including those involving children, further emphasizing the pervasive brutality [6]. In addition, survivors of sexual and gender-based violence, often cannot access post-rape care within the critical 72-hour window, exacerbating their physical and emotional trauma and health risks.

## Health systems and services in collapse

Conflict exacerbates vulnerabilities for women, newborns, and children, both during active hostilities and in the prolonged recovery periods that follow, leaving millions without access to essential care. Pediatric and neonatal care are particularly vulnerable, as conflict erodes the capacity to manage infections, prematurity, and birth complications. Damaged infrastructure, displaced health workers, and supply chain disruptions limit access to life-saving services, including emergency obstetric and newborn care. Beyond this, women are being forced to carry pregnancies to term due to limited access to contraceptives, family planning and safe abortion care [7]. As a result, the 25 countries identified in the 2024 UN Humanitarian Appeal account for 58% of global maternal deaths, 38% of newborn deaths, and 36% of stillbirths [8].

Yet, there is hope. We can learn from previous practices and the increasing body of knowledge on effective approaches and evidence-based solutions to upholding critical health services and protecting vulnerable women and children in these settings.

## Moving towards evidence-based solutions

Addressing the persistent disruptions of sexual, reproductive, maternal, newborn and child health services in conflict and post-conflict settings requires a coordinated, evidence-based approach that prioritizes resilience, community ownership, integration and sustainability. We argue that these challenges require a multi-faceted approach addressing the scope and complexity of the drivers of mortality, along with a contextualized agenda and locally adapted responses. Strengthening health systems to be adaptive and resilient through emergency preparedness is fundamental to delivering comprehensive SRMNCAH services in humanitarian and fragile settings, addressing the inequitable burden of mortality and morbidity on women, children, and adolescents [9, 10].

## Strengthening coordination

Effective coordination among implementing agencies and first-line respondents, including healthcare providers, supported by UN-led clusters, is vital for sustaining healthcare delivery during conflicts. Good practices are emerging of effective coordination between NGOs, governments, international organizations and multisector stakeholders on health service delivery within conflicts [11]. Evidence also suggests that health systems inputs and data collection were positively impacted from coordinated partner efforts [11, 12]. In addition, strategic planning helps address governance gaps caused by crises, ensuring smooth transitions between emergency response and recovery.

## Crisis-specific services and interventions

Prioritization of training and the development of toolkits is needed to optimize the implementation of the Minimum Initial Service Package for Reproductive Health in crisis settings, alongside other tailored resources to improve care for vulnerable women, newborn and children [13]. Ensuring that practice-related guidance is adapted to address crisis-specific challenges is vital, such as reliance on non-specialist staff, shortages of standard medical equipment, frequent stock-outs of essential medicines, and unreliable power and water supplies.

## Protecting the health workforce

Safeguarding health workers, particularly women, from conflict-related violence is essential to sustaining healthcare delivery in crisis settings and providing essential services to vulnerable communities. Focus must remain on evidence-based approaches for implementing context-specific policies—such as fair and appropriate incentives, security protocols, data protection measures, flexible working arrangements, safe rooms, personal protective equipment, and psychosocial support. In many contexts, identifying and supporting women leaders within communities has been necessary to support women’s health needs and improve health outcomes from mothers and newborns [14]. Engaging local communities to strengthen healthcare systems further ensures resilience and trust. Prioritizing the hiring and retention of local professionals, in conjunction with consultation with local leaders and elders, builds community trust, ensures cultural appropriateness, enhances sustainability and builds accountability from within the community to stay commitment to RMNCAH health service delivery [14].

## Alignment with local needs

Local ownership of health service delivery supports adaptive and people-centered care, enhances trust in healthcare systems, fosters community resilience, and allows for tailored responses to crises. By building the capacity of local actors, including civil society organizations, and ensuring their active participation in decision-making, interventions become more aligned with local needs and priorities [15]. This approach not only strengthens health systems during emergencies but also lays the foundation for long-term recovery and self-reliance.

**Fig 1:**
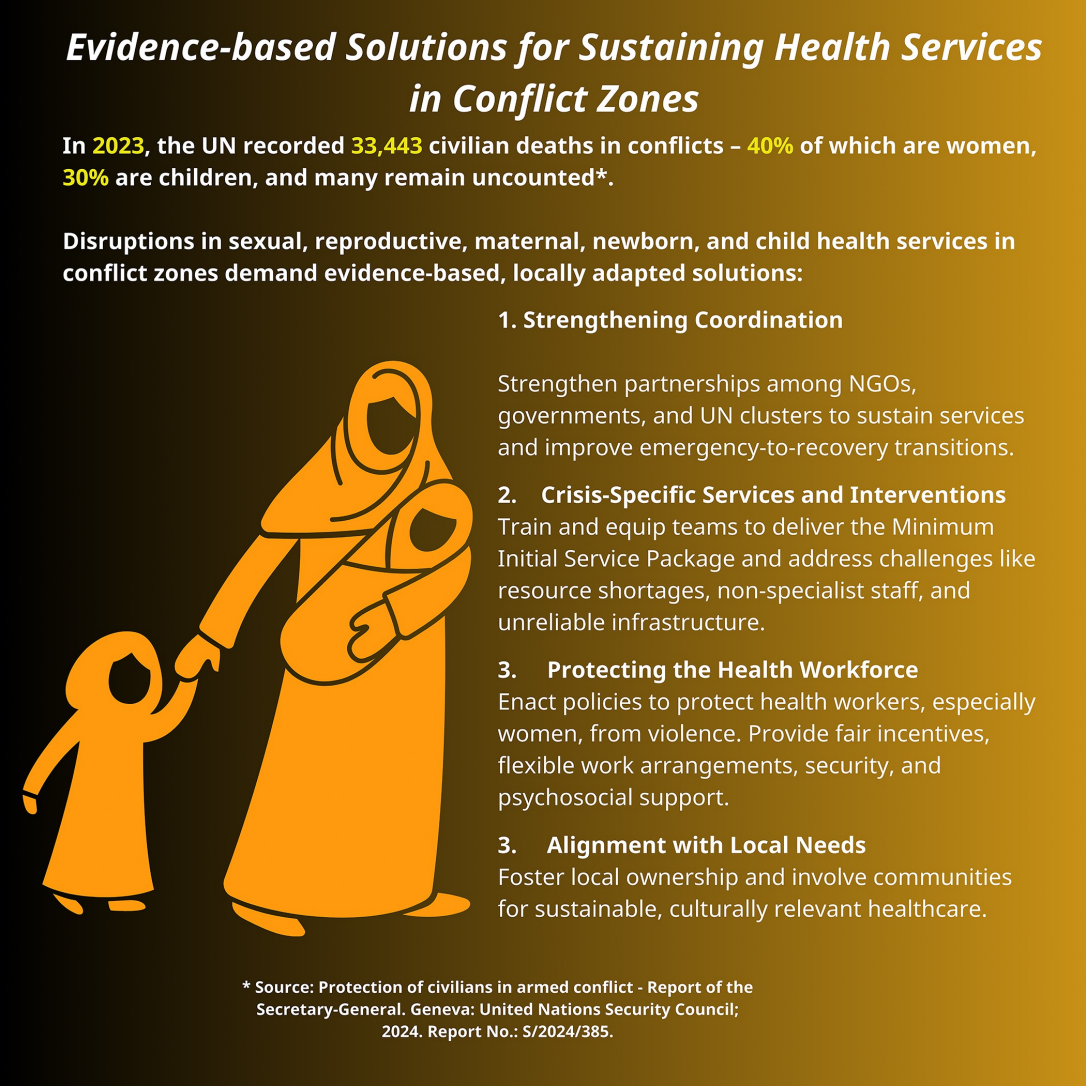
Evidence-based solutions for sustaining health services in conflict zones [2].

Ensuring the health and dignity of women, newborns, and children in conflict settings is important to fostering resilience and rebuilding communities in the face of adversity. By protecting healthcare workers and facilities, ensuring coordination among stakeholders, and securing sustainable funding, we can deliver timely and effective care. Evidence-based solutions, guided by the voices of affected communities, are essential to building resilient health systems that endure beyond conflict (Fig 1). The protection of the vulnerable must remain a non-negotiable priority in the face of global instability.
